# Antidepressant use and risk of epilepsy and seizures in people aged 20 to 64 years: cohort study using a primary care database

**DOI:** 10.1186/s12888-015-0701-9

**Published:** 2015-12-17

**Authors:** Trevor Hill, Carol Coupland, Richard Morriss, Antony Arthur, Michael Moore, Julia Hippisley-Cox

**Affiliations:** 1 Division of Primary Care, University of Nottingham, 13th floor, Tower Building, University Park, Nottingham, NG7 2RD UK; 2 Institute of Mental Health, Jubilee Campus, University of Nottingham Innovation Park, Triumph Road, Nottingham, NG7 2TU UK; 3 School of Health Sciences, Faculty of Medicine and Health Sciences, Edith Cavell Building, University of East Anglia, Norwich Research Park, Norwich, NR4 7TJ UK; 4 University of Southampton Faculty of Medicine, Primary Care and Population Sciences, Aldermoor Health Centre, Aldermoor Close, Southampton, SO16 5ST UK

**Keywords:** Antidepressants, SSRI, TCA, Depression, Epilepsy, Seizures, Primary care database, Survival analysis

## Abstract

**Background:**

Epilepsy is a serious condition which can profoundly affect an individual’s life. While there is some evidence to suggest an association between antidepressant use and epilepsy and seizures it is conflicting and not conclusive. Antidepressant prescribing is rising in the UK so it is important to quantify absolute risks with individual antidepressants to enable shared decision making with patients. In this study we assess and quantify the association between antidepressant treatment and the risk of epilepsy and seizures in a large cohort of patients diagnosed with depression aged between 20 and 64 years.

**Methods:**

Data on 238,963 patients with a diagnosis of depression aged 20 to 64 from 687 UK practices were extracted from the QResearch primary care database. We used Cox’s proportional hazards to analyse the time to the first recorded diagnosis of epilepsy/seizures, excluding patients with a prior history and estimated hazard ratios for antidepressant exposure adjusting for potential confounding variables.

**Results:**

In the first 5 years of follow-up, 878 (0.37 %) patients had a first diagnosis of epilepsy/seizures with the hazard ratio (HR) significantly increased (*P* < 0.01) for all antidepressant drug classes and for 8 of the 11 most commonly prescribed drugs. The highest risks (in the first 5 years) compared with no treatment were for trazodone (HR 5.41, 95 % confidence interval (CI) 3.05 to 9.61, number needed to harm (NNH) 65), lofepramine (HR 3.09, 95 % CI 1.73 to 5.50, NNH 138), venlafaxine (HR 2.84, 95 % CI 1.97 to 4.08, NNH 156) and combined antidepressant treatment (HR 2.73, 95 % CI 1.52 to 4.91, NNH 166).

**Conclusions:**

Risk of epilepsy/seizures is significantly increased for all classes of antidepressant. There is a need for individual risk-benefit assessments in patients being considered for antidepressant treatment, especially those with ongoing mild depression or with additional risk factors. Residual confounding and indication bias may influence our results, so confirmation may be required from additional studies.

**Electronic supplementary material:**

The online version of this article (doi:10.1186/s12888-015-0701-9) contains supplementary material, which is available to authorized users.

## Background

In the UK, antidepressant prescribing has been rising in recent years [1]; in 2012 more than 50 million antidepressant prescriptions were dispensed in England alone [2]. There is some evidence that treatment with antidepressants is associated with an increased risk of epilepsy and seizures [3, 4], however few long-term studies have been carried out in the general population. Epilepsy affects more than 500,000 people in the UK, meaning around 1 in 100 suffer from the condition [5].

Epilepsy is not only a serious and debilitating disease, with treatment which can be quite problematic [6], but individuals with epilepsy are more likely to suffer from several psychiatric disorders, including major depression [7]. In addition there is evidence of a bidirectional association between depression and epilepsy/seizures [8]. In a previous study [4, 9] of patients with depression over the age of 65, we found that certain antidepressants were associated with an increased risk of epilepsy/seizures. Large scale primary care database studies provide the opportunity to test whether there are clinically important differences between individual antidepressants and the risk of epilepsy/seizures. Furthermore they provide an opportunity to calculate absolute risk that can be considered alongside other benefits and risks of prescribing antidepressants. We therefore carried out a study to look at the risk of epilepsy/seizures in adults aged 20 to 64 years taking antidepressants. We aimed to assess and quantify the change in absolute risk of epilepsy/seizures associated with antidepressant use, in a population with no known predisposition to epilepsy or seizures.

## Methods

The cohort study was designed to assess and quantify associations with antidepressant treatment for a number of different safety outcomes including epilepsy/seizures. Full details of the study design and methods can be found in the published study protocol [10].

### Study cohort

Patient data from 687 general practices across the UK was extracted from the QResearch primary care database (version 34), a large repository of anonymised health records for more than twelve million patients registered with general practices using the Egton Medical Information System (EMIS).

Patients were selected for the cohort if they had a first recorded diagnosis of depression between the ages of 20 to 64, and within the study period (1/1/2000 to 31/7/2011). To allow for at least 12 months of follow-up the study end date was set to 1/8/2012. Identification of patients with a diagnosis of depression was achieved using clinical Read codes that have been used in previous studies [4, 11]. Depression in the UK is largely diagnosed and treated in primary care without the input of specialist assessment and there is no widespread formal screening for depression using validated tools.

The study entry date for each patient was defined as either: the date of their first recorded diagnosis of depression, or the date of their first antidepressant prescription if that occurred before the diagnosis date and within 36 months of diagnosis. To ensure only patients with a first diagnosis of depression were included in the cohort, eligible patients had to be diagnosed with depression at least 12 months after registering with the practice and after the date the EMIS system was installed.

Patients with a recorded diagnosis of depression before the study entry date and those prescribed antidepressants before this date, before their date of registration with the practice, before the age of 20 or more than 36 months before their first recorded diagnosis of depression were excluded. Temporary residents, patients with diagnoses of schizophrenia, bipolar disorder or other types of psychosis, or those prescribed lithium or anti-manic drugs at the study entry date were also excluded. In addition, although we have included the number of patients receiving MAOI prescriptions, and the number with a previous history of epilepsy/seizures in descriptive results, before proceeding with the main analyses, patients satisfying either of these criteria were dropped from the cohort.

Follow-up continued until the earliest date of: the patient’s first recorded diagnosis of epilepsy/seizures, the patient left the practice, death, or the end of the follow-up period (1/8/2012). This became the patient’s study exit date.

### Outcomes

The outcome was the first recorded diagnosis of epilepsy or a seizure in the patients’ primary care medical record. This outcome was identified using 88 separate Read codes for epilepsy/seizures (see Additional file 1: Table S1).

### Exposure data

Data on antidepressant prescriptions during follow-up were extracted, including the date of each prescription, the antidepressant drug name, the dose of each tablet in milligrams, dosage instructions, plus the number of tablets or capsules prescribed. The duration in days for each prescription was calculated by dividing the number of tablets prescribed by the number to be taken per day indicated by the dosage instructions. Where dosage directions were missing or unclear (just under 5 % of prescriptions) we used the median duration from prescriptions for the same drug where dosage was recorded, accounting for the number of tablets prescribed, as in our previous study [4, 9]. The total daily dose was generated by multiplying the dose per tablet by the number of tablets to be taken each day. In order to make direct comparisons between different antidepressant classes we converted the total daily dose to a defined daily dose (DDD) using the assumed average daily maintenance dose as given for each antidepressant on the WHO website (www.whocc.no/atc_ddd_index/). Where prescriptions for the same drug were issued on the same day we counted them as a single prescription and summed the doses.

We classified antidepressant drugs according to the classes in the British National Formulary (BNF) which gives four main categories: tricyclic and related antidepressants (TCAs - section 4.3.1), selective serotonin reuptake inhibitors (SSRIs - section 4.3.3), monoamine oxidase inhibitors (MAOIs - section 4.3.2), and other antidepressants (section 4.3.4). We defined an additional fifth category as combined prescriptions, comprising prescriptions for different drugs, either within a class or from different classes, given on the same date; these prescriptions were not included in analyses of dose.

In a similar way to the previous study [4, 9] the 11 most frequently prescribed individual antidepressants were identified.

### Confounding variables

Both demographic and baseline characteristics and comorbidities were included as confounding factors in the adjusted analyses. These were a general pool of confounders considered likely to be associated with either the selection of an antidepressant or the risk of the outcome as in the previous study [9].

The demographic features were: age at study entry, gender, year of index diagnosis of depression, severity of index diagnosis of depression (categorised as mild, moderate or severe, using the Read code classification published by Martinez and colleagues [11] and some additional classification by a member of the study team (RM) of further Read codes for depression, present in our data but not included in the Martinez study, see Additional file 1: Table S2), Townsend deprivation score (in fifths) [12], smoking status (non-smoker, ex-smoker, current smoker coded as light (1–9 cigarettes/day), moderate (10–19 cigarettes/day) or heavy (≥20 cigarettes/day)), alcohol intake (none, trivial (less than 1 unit per day), light (1 to 2 units per day), medium (3 to 6 units per day), heavy (7 to 9 units per day) and very heavy (more than 9 units per day)), and ethnicity categorised as either white/not recorded or non-white (comprising Indian, Pakistani, Bangladeshi, other Asian, black African, black Caribbean, Chinese and other/mixed groups).

We also adjusted for 13 baseline comorbidities and for usage of 11 drugs at baseline, including anticonvulsants. A full list of these can be found in Table 1.

**Table 1:** Characteristics of the study cohort (*N* = 238,963) at baseline

Characteristic		n	%
Gender			
	Male	92,935	38.89
	Female	146,028	61.11
Age (years)			
	20-29	51,212	21.43
	30-39	77,141	32.28
	40-49	59,260	24.80
	50-59	39,573	16.56
	60-64	11,777	4.93
Mean age (SD)			
	Overall	39.54 (11.14)	
	Male	40.98 (11.13)	
	Female	38.61 (11.04)	
Ethnicity			
	White/not recorded	227,451	95.18
	Indian	1922	0.80
	Pakistani	1714	0.72
	Bangladeshi	1000	0.42
	Other Asian	991	0.41
	Caribbean	1520	0.64
	Black African	1386	0.58
	Chinese	307	0.13
	Other/mixed	2672	1.12
Depression severity (index diagnosis)			
	Mild	171,208	71.65
	Moderate	59,140	24.75
	Severe	8615	3.61
Perinatal depression^a^		18,259	12.50
Smoking^b^			
	Recorded	233,290	97.63
	Non smoker	110,849	47.52
	Ex smoker	35,132	15.06
	Current smoker	87,309	37.43
	Light smoker (1–9 per day)	24,104	10.33
	Moderate smoker (10–19 per day)	40,546	17.38
	Heavy smoker (≥20 per day)	22,659	9.71
Alcohol consumption^b^			
	Recorded	203,189	85.03
	Non drinker	55,253	27.19
	Trivial (less than 1 unit per day)	77,579	38.18
	Light (1–2 units per day)	51,310	25.25
	Moderate (3 to 6 units per day)	14,482	7.13
	Heavy (7 to 9 units per day)	2174	1.07
	Very heavy (over 9 units per day)	2391	1.18
Townsend deprivation quintile^b^			
	Recorded	230,762	96.57
	1 (Least deprived)	45,021	19.51
	2	46,207	20.02
	3	48,293	20.93
	4	47,063	20.39
	5 (Most deprived)	44,178	19.14
Comorbidities			
	CHD	4109	1.72
	Diabetes	7371	3.08
	Hypertension	17,217	7.20
	Stroke/TIA	1741	0.73
	Cancer	3810	1.59
	Epilepsy/seizures	3325	1.39
	Hypothyroidism	5267	2.20
	Obsessive-compulsive disorder	494	0.21
	Rheumatoid arthritis	1301	0.54
	Osteoarthritis	7228	3.02
	Osteoporosis	867	0.36
	Liver disease	698	0.29
	Renal disease	549	0.23
	Asthma/chronic obstructive airways disease	31,816	13.31
Medications at baseline			
	Anticonvulsants	2672	1.12
	Antihypertensives	25,344	10.61
	Anti-psychotics	836	0.36
	Aspirin	7159	3.00
	Anticoagulants	1073	0.45
	Bisphosphonates	854	0.36
	Hypnotics/anxiolytics	11,354	4.75
	NSAIDs	12,725	5.33
	Statins	10,823	4.53
	Oral contraceptives^a^	27,396	18.76
	HRT^a^	7207	4.94

^a^ Given percentage is for female patients only

^b^ Percentages are out of the number recorded

### Statistical analysis

We used Cox’s proportional hazards models to analyse the time to the first recorded diagnosis of epilepsy/seizures and to assess any differences in risk associated with the antidepressant prescribed. These analyses used robust standard errors to account for clustering of patients within general practices. Patients with a previous history of epilepsy/seizures were dropped from the cohort before proceeding with these analyses. Our analysis treated antidepressant use as a time-varying exposure which partitioned follow-up time for each patient into treatment periods by antidepressant class and also any periods of non-treatment between prescriptions. We regarded a patient as not exposed to treatment during periods where there was a gap of 90 days or more between the end of one prescription and the start of the next. We based our main results on the first 5 years of follow-up after study entry, and also conducted analyses for the first year of follow-up and for the whole of follow-up time. We calculated unadjusted and adjusted hazard ratios with 95 % confidence intervals, adjusting for the confounding variables listed above.

The analysis of antidepressant treatment was conducted in five main ways: by drug class (with no current treatment as the reference group), drug class (with SSRIs as the reference group), dosage, the 11 most common antidepressants, and by time since starting and stopping antidepressant treatment. Table 3 shows the categories used. For the dosage analysis we did not calculate DDDs for combined prescriptions, so only used one category for them. For the time since starting and stopping analysis, periods of non-treatment from 183 days after stopping treatment were coded as no use of antidepressants.

We tested for interactions between antidepressant drug class and age, sex and ethnicity.

We determined statistical significance using a two-tailed *P* value of <0.01, and used Wald’s significance tests to assess differences between drug classes. We used log-log plots and tests of the proportional hazards assumption via Schoenfeld residuals, to check the validity of our models.

Numbers needed to harm (NNH) and absolute risks were calculated over 1 and 5 years based on the method described for numbers needed to treat (NNT) by Altman et al. [13, 14] using adjusted hazard ratios. The NNH for a particular drug gives the number of patients that would need to be treated with that drug, over a certain period of time, for there to be one extra case of epilepsy/seizures more than that occurring in the untreated, comparison group. The quantity is generated by first calculating the absolute risk increase by subtracting the event rate in the treated group from that in the untreated control group (at a specific time), and then taking the reciprocal of that number. The NNH assumes a causal relationship between each drug and the risk of epilepsy/seizures.

All analyses were conducted with Stata/MP v12.1.

### Sensitivity analyses

We performed two sensitivity analyses. In the first we repeated the main analyses, but excluded patients who received no antidepressant treatment during follow-up. The reason for this analysis was to examine whether any differences between the untreated and treated patients that we were not able to account for in the analysis, such as a preference for psychotherapy rather than drug treatment, could introduce a form of selection bias into our results.

In the second we repeated the analyses excluding patients on anticonvulsants at baseline, in case these had a previous history of epilepsy that was unrecorded and could bias the results, although anticonvulsant use is not necessarily an indication of epilepsy or seizures as these drugs are sometimes used in the treatment of other conditions such as neuropathic pain [15].

### Approvals

The project has been independently peer reviewed and accepted by the QResearch Scientific board and has been approved in accordance with the agreed procedure with the Trent Research Ethics Committee (reference No MREC/03/4/021).

## Results

Our initial cohort consisted of 327,235 patients aged 20 to 64 years with a first diagnosis of depression recorded between 1st January 2000 and 31st July 2011, and excluded temporary residents and those with a previously recorded diagnosis of depression. We excluded a further 88,272 (27.0 %) patients. Of these, 7152 (2.2 %) patients were excluded who had a diagnosis of schizophrenia, bipolar disorder or another psychosis. Also 83,824 (25.6 %) patients were excluded who either were prescribed antidepressant medication more than 36 months before the recorded date of depression diagnosis, before their study entry date, or before the age of 20. These criteria were not mutually exclusive and there was some overlap between them. The number of patients in the study cohort became 238,963 (73 % of the initial sample).

### Demographic characteristics of the cohort

Table 1 shows the baseline characteristics of the cohort. There were 146,028 (61.1 %) females and 92,935 (38.9 %) males. The mean age was 39.5 years (SD = 11.1).

The most common comorbidity at baseline was asthma and/or chronic obstructive airways disease (31,816 patients, 13.3 %), followed by hypertension (17,217 patients, 7.2 %). The most common medication at baseline was antihypertensives (25,344 patients, 10.6 %). There were 2672 (1.1 %) patients on anticonvulsants at baseline.

### Antidepressant treatment during follow-up

A total of 209,476 (87.6 %) patients received antidepressant treatment during follow-up, with 3,337,336 antidepressant prescriptions issued during the whole of follow-up. This mainly comprised SSRI prescriptions (2,379,668, 71.3 %), with 533,798 (16.0 %) prescriptions for TCAs, 422,079 (12.7 %) for other types of antidepressant and only 1791 (<0.1 %) prescriptions for MAOIs. The median duration of treatment during follow-up was 221 days (interquartile range 79 to 590 days), with 5.5 % of patients having a total duration of treatment of 5 or more years. Within the whole study period there were over 1.3 million person-years of follow-up.

There were 168,457 prescriptions for two or more different antidepressants issued on the same date. After counting these as single combined prescriptions the total number of prescriptions became 3,252,633. Table 2 gives information on the most frequently prescribed individual antidepressants. The most frequently prescribed antidepressant was citalopram with 1,023,255 (31.5 %) prescriptions, followed by fluoxetine with 778,285 (23.9 %) prescriptions.

**Table 2:** Number of prescriptions, DDD and doses prescribed for the 11 most commonly prescribed antidepressant drugs

				Dose prescribed (mg/day)^c^				
Antidepressant drug	n^a^	%^b^	DDD	Mode	Median	IQR	Min	Max
*Tricyclic and related antidepressants (TCA)*								
Amitriptyline hydrochloride	236,416	7.27	75	25	25	15 to 50	2.0	350.0
Dosulepin hydrochloride	125,302	3.85	150	75	75	50 to 150	12.5	325.0
Lofepramine	47,414	1.46	105	140	140	140 to 210	35.0	350.0
Trazodone hydrochloride	30,912	0.95	300	150	125	75 to 150	20.0	750.0
*Selective serotonin reuptake inhibitors (SSRI)*								
Citalopram hydrobromide	1,023,255	31.46	20	20	20	20 to 20	2.5	120.0
Escitalopram	139,190	4.28	10	10	10	10 to 20	2.5	80.0
Fluoxetine hydrochloride	778,285	23.93	20	20	20	20 to 20	4.0	120.0
Paroxetine hydrochloride	159,389	4.90	20	20	20	20 to 30	4.0	100.0
Sertraline hydrochloride	213,749	6.57	50	50	50	50 to 100	12.5	400.0
*Other antidepressants*								
Mirtazapine	142,400	4.38	30	30	30	15 to 45	3.8	112.5
Venlafaxine hydrochloride	205,984	6.33	100	75	112.5	75 to 150	9.4	600.0
All other antidepressants	66,553	2.05						
Combined antidepressants	83,784	2.58						

*DDD* defined daily dose

^a^Numbers of prescriptions where prescriptions for the same drug issued on the same day count as a single prescription and the doses have been summed

^b^Percentage out of total number of prescriptions = 3,252,633

^c^4.98 % of prescriptions had missing information on dosage

See Additional file 1: Table S3 for a breakdown of baseline characteristics for the cohort by the type of antidepressant first prescribed, including a column for those patients not treated with antidepressants during follow-up.

### Number of cases of epilepsy/seizures at baseline and during follow-up

At baseline 3325 patients (1.4 %) had a previous diagnosis of epilepsy and/or seizures. We excluded these patients and also, due to small numbers, those with MAOI prescriptions (*n* = 156) from further analyses leaving 235,489 patients (72 % of the initial sample).

During the entire follow-up period 1126 patients had a first diagnosis of epilepsy/seizures. Within the first 5 years of follow-up 878 patients (416 women and 462 men) had a first diagnosis of epilepsy/seizures, giving an incidence rate of 99 per 100,000 person-years (76 in women and 135 in men).

### Associations with epilepsy/ seizures over 5 years of follow-up

Table 3 shows unadjusted and adjusted hazard ratios for the first 5 years of follow up. All hazard ratios for the different antidepressant drug classes were statistically significant compared with periods of no use, with the highest being for combined antidepressants (adjusted HR 2.73, 95 % CI 1.52 to 4.91). There was no significant difference between the drug classes overall (*p* = 0.254).

**Table 3:** Unadjusted and adjusted 5-year hazard ratios for epilepsy/seizures by antidepressant class, dose, duration and individual drug

	No. of events^a^	Person years^b^	Unadjusted analyses				Adjusted^c^ analyses			
			HR	95 % CI		P	HR	95 % CI		P
Antidepressant class										
No current use	384	566,890	1.00				1.00			
TCAs	82	41,130	2.83	2.22	3.62	<0.001	2.32	1.79	3.01	<0.001
SSRIs	309	224,600	1.97	1.69	2.31	<0.001	1.92	1.63	2.25	<0.001
Other antidepressants	58	27,820	3.11	2.39	4.05	<0.001	2.33	1.76	3.10	<0.001
Combined antidepressants	13	4220	4.70	2.77	7.98	<0.001	2.73	1.52	4.91	0.001
Antidepressant class and dose categories^d^										
No current use	384	566,890	1.00				1.00			
TCA: ≤ 0.5 DDD	38	23,520	2.26	1.59	3.21	<0.001	1.91	1.32	2.76	0.001
TCA: >0.5 DDD/≤ 1.0 DDD	28	8370	4.85	3.31	7.11	<0.001	3.65	2.46	5.42	<0.001
TCA: > 1.0 DDD	14	5240	3.69	2.18	6.24	<0.001	2.94	1.72	5.03	<0.001
SSRI: ≤ 0.5 DDD	19	15,970	1.67	1.06	2.61	0.027	1.80	1.15	2.84	0.011
SSRI: >0.5 DDD/≤ 1.0 DDD	213	157,490	1.92	1.60	2.30	<0.001	1.90	1.57	2.29	<0.001
SSRI: > 1.0 DDD	67	42,410	2.31	1.80	2.97	<0.001	2.03	1.56	2.64	<0.001
Other: ≤ 0.5 DDD	6	4000	2.09	0.95	4.61	0.068	1.71	0.77	3.82	0.187
Other: >0.5 DDD/≤ 1.0 DDD	31	13,090	3.71	2.64	5.20	<0.001	2.66	1.85	3.83	<0.001
Other: > 1.0 DDD	19	8330	3.21	2.01	5.13	<0.001	2.54	1.56	4.11	<0.001
Combined antidepressants	13	4220	4.70	2.77	7.97	<0.001	2.73	1.52	4.91	0.001
Antidepressant class by duration										
No current use^e^	333	508,970	1.00				1.00			
TCA first 28 days	12	5470	2.85	1.52	5.36	0.001	2.40	1.28	4.47	0.006
TCA 29 to 84 days	16	5380	4.65	2.86	7.59	<0.001	3.76	2.29	6.18	<0.001
TCA 85 or more days	36	18,900	2.86	2.02	4.05	<0.001	2.16	1.49	3.14	<0.001
SSRI first 28 days	27	20,590	1.67	0.94	2.95	0.079	1.61	0.92	2.83	0.098
SSRI 29 to 84 days	36	27,800	2.14	1.46	3.12	<0.001	2.03	1.39	2.97	<0.001
SSRI 85 or more days	185	127,070	2.17	1.81	2.61	<0.001	2.07	1.70	2.51	<0.001
Other first 28 days	10	2750	4.96	2.58	9.53	<0.001	3.93	2.04	7.56	<0.001
Other 29 to 84 days	3	3480	1.71	0.64	4.57	0.283	1.02	0.33	3.21	0.968
Other 85 or more days	30	16,740	2.78	1.96	3.95	<0.001	2.03	1.41	2.94	<0.001
Combined, all time	13	3850	5.35	3.15	9.09	<0.001	2.99	1.65	5.40	<0.001
TCA 1–28 days after stopping	3	3610	1.28	0.41	3.95	0.668	1.14	0.37	3.54	0.818
TCA 29–84 days after stopping	15	7020	3.12	1.86	5.24	<0.001	2.79	1.65	4.72	<0.001
TCA 85–182 days after stopping	8	10,690	1.30	0.69	2.45	0.425	0.95	0.47	1.92	0.880
SSRI 1–28 days after stopping	23	15,640	2.24	1.47	3.39	<0.001	2.28	1.50	3.46	<0.001
SSRI 29–84 days after stopping	33	30,320	1.66	1.14	2.44	0.009	1.60	1.07	2.37	0.021
SSRI 85–182 days after stopping	41	46,690	1.31	0.94	1.81	0.109	1.25	0.89	1.76	0.197
Other 1–28 days after stopping	3	1560	2.86	0.93	8.79	0.067	2.39	0.77	7.39	0.131
Other 29–84 days after stopping	11	2990	5.79	3.27	10.28	<0.001	4.46	2.46	8.09	<0.001
Other 85–182 days after stopping	8	4490	2.52	1.25	5.08	0.010	2.17	1.08	4.36	0.030
Combined 1–182 days after stopping^f^	0	660	n/a	-	-	-	n/a	-	-	-
Antidepressant drug										
No current use	384	566,890	1.00				1.00			
Amitriptyline (TCA)	34	19,550	2.46	1.75	3.46	<0.001	1.94	1.35	2.78	<0.001
Dosulepin (TCA)	21	12,120	2.55	1.63	3.97	<0.001	2.19	1.38	3.47	0.001
Lofepramine (TCA)	12	4750	3.48	1.97	6.14	<0.001	3.09	1.73	5.50	<0.001
Trazodone (TCA)	12	2320	7.20	4.08	12.71	<0.001	5.41	3.05	9.61	<0.001
Citalopram (SSRI)	136	93,940	2.07	1.71	2.52	<0.001	2.03	1.66	2.49	<0.001
Escitalopram (SSRI)	14	13,310	1.47	0.83	2.58	0.185	1.49	0.84	2.63	0.171
Fluoxetine (SSRI)	111	81,780	1.94	1.55	2.42	<0.001	1.92	1.52	2.41	<0.001
Paroxetine (SSRI)	26	16,500	2.35	1.50	3.67	<0.001	2.02	1.27	3.23	0.003
Sertraline (SSRI)	22	18,790	1.71	1.12	2.61	0.013	1.56	1.01	2.42	0.045
Mirtazapine (other)	17	10,070	2.35	1.47	3.78	<0.001	1.72	1.06	2.80	0.028
Venlafaxine (other)	36	15,500	3.61	2.59	5.02	<0.001	2.84	1.97	4.08	<0.001
All other antidepressants	8	4910	2.30	1.16	4.54	0.017	1.67	0.84	3.33	0.143
Combined antidepressants	13	4220	4.70	2.77	7.98	<0.001	2.71	1.50	4.87	0.001

*DDD* defined daily dose

^a^Based on numbers in adjusted analysis. For the first 5 years of follow-up there were 878 cases of epilepsy/seizures in total, and 1126 in total for the whole of follow-up. The total number of cases here is less than 878 due to some being dropped due to missing data on covariates

^b^Person years of exposure, based on adjusted analyses

^c^Adjusted for age, sex, year of diagnosis of depression, severity of depression, deprivation, smoking status, alcohol intake, ethnic group (white/not recorded or non-white), coronary heart disease, diabetes, hypertension, cancer, hypothyroidism, osteoarthritis, asthma/chronic obstructive airways disease, stroke/TIA, rheumatoid arthritis, osteoporosis, liver disease, renal disease, obsessive-compulsive disorder, statins, NSAIDS, aspirin, antihypertensive drugs, anticonvulsants, hypnotics/anxiolytics, oral contraceptives, hormone replacement therapy, antipsychotics, bisphosphonates, anticoagulants

^d^Total numbers in the analysis of dosage are less due to missing data on dose

^e^Number of events in the no treatment group is less due to some now being included in the time since stopping categories

^f^There were no events in the 1–182 days after stopping for combined antidepressants

All dose categories other than low doses of SSRIs and other antidepressants were associated with a significantly increased risk (at *P* < 0.01) of epilepsy/seizures compared to periods of no treatment. Hazard ratios increased with dose for SSRIs but for TCAs and other antidepressants the middle dose categories had the highest hazard ratios.

There were significantly increased hazard ratios for 8 of the 11 most commonly prescribed drugs with the highest risks associated with trazodone (adjusted HR = 5.41, 95 % CI 3.05 to 9.61), lofepramine (adjusted HR = 3.09, 95 % CI 1.73 to 5.50), and venlafaxine (adjusted HR = 2.84, 95 % CI 1.97 to 4.08). Escitalopram, sertraline and mirtazapine, were not associated with a significant increase in risk (at *P* < 0.01). Wald’s tests however did not indicate any significant difference overall between all the individual antidepressant drugs (*P* = 0.029).

When compared against the drug citalopram, only trazodone (HR = 2.66, 95 % CI 1.49 to 4.76) had a significantly increased risk of epilepsy/seizures. Full results of analyses of direct comparisons with mid-dose SSRIs and citalopram are shown in Additional file 1: Table S4.

**Fig. 1:**
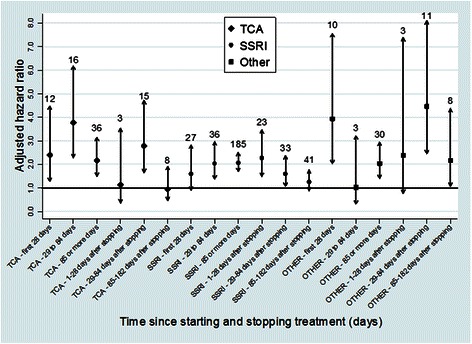
Adjusted hazard ratios and 95 % confidence intervals for epilepsy/seizures, for time since starting and stopping treatment by drug class over 5 years of follow-up. Note: numbers above each arrow are the number of cases of epilepsy/seizures in that category

In the analysis of duration (Table 3 and Fig. 1), there were no clear patterns of risk and all hazard ratios were significant except for SSRIs in the first 28 days of treatment and other antidepressants in the 29–84 days category. In the time since stopping treatment categories only 3 were statistically significant: the first 28 day period for SSRIs (adjusted HR 2.28, 95 % CI 1.50 to 4.36) and the 29–84 day periods for TCAs (adjusted HR 2.79, 95 % CI 1.65 to 4.72), and other antidepressants (adjusted HR 4.46, 95 % CI 2.46 to 8.09). However numbers were small in some categories.

### Interaction models

There were no statistically significant interactions between drug class and either age, sex or ethnicity.

### Sensitivity analyses

In the first sensitivity analysis excluding untreated patients, although all hazard ratios increased slightly, only one result, for low dose SSRI, went from borderline significance to statistical significance (HR from 1.80 to 1.88, with P moving from 0.011 to 0.006) (Additional file 1: Table S5).

In the second sensitivity analysis omitting patients on anticonvulsants at baseline (Additional file 1: Table S6), most hazard ratios increased with the significance levels largely remaining unchanged. For individual drugs the only hazard ratio to decrease was for escitalopram, going from 1.49 to 1.33. The greatest increase was for trazodone (HR from 5.41 to 5.95).

### Associations with epilepsy/seizures over 1 year’s follow-up

We repeated the analyses restricted to the first year of follow-up and the hazard ratios tended to be lower and with wider confidence intervals due to the reduced number of events (Additional file 1: Tables S7 and S8).

### Absolute risks of epilepsy/seizures

Numbers needed to harm (NNH) and absolute risks over one and five years of follow-up are shown in Table 4. These assume a causal association, and confidence intervals are only shown for the drugs with statistically significant associations with epilepsy/seizure risk based on the adjusted hazard ratios.

**Table 4:** Numbers needed to harm (NNH) for epilepsy/seizures, at 1-year and 5-years of follow-up for drug class and individual antidepressant

	1 year follow-up			5 years follow-up		
	Absolute risk per 1000^a^	NNH	95 % CI^b^	Absolute risk per 1000^a^	NNH	95 % CI^b^
TCA	1.6	1437	609 to 9786	8.1	217	143 to 363
SSRI	1.4	2014	1014 to 7458	6.7	312	229 to 453
Other	1.8	1125	472 to 6274	8.2	215	137 to 378
Combined	5.5	217	82 to 705	9.5	166	74 to 551
*Tricyclic and related antidepressants (TCA)*						
Amitriptyline	1.7	1332	-	6.8	306	161 to 819
Dosulepin	1.5	1572	-	7.6	241	117 to 748
Lofepramine	1.3	2565	-	10.8	138	64 to 392
Trazodone	2.6	602	-	18.8	65	34 to 140
*Selective serotonin reuptake inhibitors (SSRI)*						
Citalopram	1.8	1126	614 to 2745	7.1	278	193 to 434
Escitalopram	1.2	3003	-	5.2	586	-
Fluoxetine	1.3	2697	-	6.7	313	204 to 546
Paroxetine	1.0	18372	-	7.1	280	129 to 1072
Sertraline	0.7	−4684	-	5.5	508	-
*Other antidepressants*						
Mirtazapine	1.2	2962	-	6.0	396	-
Venlafaxine	2.2	749	296 to 3731	9.9	156	93 to 294
All others	1.3	2383	-	5.8	426	-
Combined	5.5	216	82 to 700	9.4	168	74 to 569

^a^Absolute risk of epilepsy/seizures in the no treatment group is 0.9 and 3.5 after 1 year and 5 years of follow-up respectively

^b^Confidence intervals are not shown where the HR is not statistically significant

The drug class with the highest NNH is the SSRIs: 2014 patients would have to be treated for a year for there to be one extra case of epilepsy/seizures. Over five years 312 patients would have to be treated for an extra case. At the other end of the spectrum, only 166 patients would have to be treated with a combined prescription for five years, for there to be an extra case. For individual drugs the lowest NNH is for trazodone, where for one extra case of epilepsy/seizures to occur 602 patients would have to be treated for one year, and only 65 patients would have to be treated for five years. Although the NNH over one year for lofepramine and venlafaxine are higher at 2565 and 749 respectively, they are only 138 and 156 for five years of follow-up. Of all the individual antidepressants, these three drugs have the lowest NNH over five years of follow-up, with trazodone having almost twice the absolute risk as the other two.

## Discussion

We have conducted a large study of primary care patients aged 20–64 years with depression and with no previous diagnosis of epilepsy/seizures. We have assessed and quantified the absolute risk of epilepsy associated for different classes and individual types of antidepressant. Our analyses have found that all classes of antidepressant were associated with a statistically significant increase in epilepsy/seizures when compared with periods of no treatment. These associations remained for all dose levels for TCAs and for all doses (other than low doses) of SSRIs and other types of antidepressant. There was also an increased risk of epilepsy/seizures in patients prescribed combined antidepressant treatment, but there were relatively few cases in this group, so the result should be treated with caution.

Of the 11 individual drugs we examined, all except sertraline, escitalopram and mirtazapine were significantly associated with the risk of epilepsy/seizures. Individual drugs with the highest risks were trazodone, lofepramine and venlafaxine. When compared against citalopram, the most commonly prescribed antidepressant, only trazodone had a significantly increased risk of epilepsy/seizures. Regarding duration of treatment, there were no clear patterns of risk although for all drug classes the risk of epilepsy/seizures remained significantly increased more than 12 weeks after starting treatment.

The statistically significant increases in risk of epilepsy/seizures described above, for all drug classes and for most dose categories and individual drugs remained largely unchanged when we omitted patients not receiving any antidepressant treatment during follow-up. When we excluded those taking anticonvulsants at baseline the hazard ratios tended to increase. This result confirms the expectation that anticonvulsants increase the fit threshold, and therefore may reduce the risk of epilepsy/seizures associated with antidepressant use. In effect, by including patients on anticonvulsants at baseline in the cohort, the hazard ratios for antidepressant use are attenuated.

Although all the drug classes and most individual drugs were associated with an increased risk of epilepsy/seizures, the NNH over the first year are quite high and mainly in the thousands, with absolute risks mostly less than 2 in 1000. Only combined prescriptions and the drugs trazodone and venlafaxine have NNH values below one thousand for the first year of treatment. Therefore in the main, for courses of antidepressant treatment that are less than one year, the absolute risk is present but low.

It is only under long term treatment, as in our example of five years that the NNH drop into the several hundred. Here, the absolute risks range from 7 per 1000 (SSRIs) to 10 per 1000 (combined) for the drug classes. For individual antidepressants, the drugs with the highest absolute risks over 5 years are: venlafaxine (10 per 1000), lofepramine (11 per 1000) and trazodone (19 per 1000). Therefore our results show that those patients receiving treatment courses in line with guidelines (around 180 days) will have a low absolute risk of epilepsy/seizures. However patients on longer courses will accumulate a moderate absolute risk, which can vary and be quite high depending on the specific drug.

### Comparison with other studies

Whilst the association between epilepsy/seizures and depression [8, 16, 17] and that between antidepressant use and epilepsy/seizures [18] has been previously documented, some of this evidence is conflicting. For example some studies claim the effects of antidepressants are anticonvulsant rather than pro-convulsant [19] and other studies claim that most if not all antidepressants lower the seizure threshold [3].

There are studies concerned only with a population already predisposed to the outcome [20–24], however their results may not be directly comparable to ours as we omitted patients with a history of epilepsy/seizures. One of these studies [20] concluded that patients could be safely treated with antidepressants from the TCA or SSRI groups without any significant increase in seizure frequency. However the study only looked at the first 12 months of treatment, only included two of the antidepressants that we did (amitriptyline and paroxetine) and was much smaller than our study (*n* = 421).

Another large study utilising drug safety data [24] concluded that SSRIs and the antidepressants mirtazapine, venlafaxine and duloxetine might be more appropriate for treating depression than TCAs, in patients with an enhanced seizure risk. They based their analysis on counting the number of grand mal seizures in patients undergoing antidepressant treatment in psychiatric inpatient settings. A limitation of this analysis is that seizures were probably underreported.

A further issue is whether the antidepressants are given in low doses, normal therapeutic doses, or taken in overdose. For example, there is evidence that citalopram has the ability to induce seizures if taken in overdose [25] and that it is more toxic and more likely to provoke seizures in overdose than escitalopram [26]. However a review by Nemeroff [27] concluded that doses of between 20–60 mg of citalopram do not increase risk of seizure.

A study utilising Food and Drug Administration (FDA) Summary Basis of Approval (SBA) Reports in the United States [28] concluded that many second-generation antidepressants, several of which are included in our study (including citalopram and venlafaxine), have anticonvulsant properties. They based their conclusions on comparing seizure incidence between treatment and placebo arms of clinical trials, with numbers of participants less than 5000 in most instances, and with short follow-up periods (the mean duration of the treatment arms was only 116 days, with the mean duration of the placebo arms only 78 days). They also found an incidence of seizures in the placebo groups approximately 19 times that seen in the general population.

These results may seem to contradict our own, but many of the placebo arms of the studies included in the Alper [28] paper did not report the number of seizures, a limitation which they acknowledge. In addition, although many of the antidepressants they looked at are also included in our study, they did not treat each antidepressant individually, but rather they combined them together and compared the overall seizure rate with the placebo arms. Also, seizures are only recorded in SBA reports if the FDA staff physician deems the event was unlikely to have been provoked by an external factor, such as alcohol withdrawal, and could be safely attributed to the drug in question. Our study included all events, without an attempt to distinguish between the possible causes. Generally clinical trials are designed to answer questions regarding benefits of treatments rather than safety and full reporting of adverse effects is often unavailable [29].

In an analysis [30] of the WHO adverse drug reactions (ADR) database, the authors concluded that antidepressant treatment, lowers the seizure threshold and provokes seizures. They looked at 9 of the 11 most commonly prescribed antidepressant medications that we did. There were some differences in their results compared to ours though, since they showed trazodone to have one of the lowest ratios of suspected seizures. There were many limitations to their study however, including the fact that the total number of patients treated with a particular drug was unknown making direct comparisons with our results inappropriate.

In our previous study in people aged 65 years and over [4, 9] we found that SSRIs and other antidepressants were associated with an increased risk of epilepsy/seizures. This situation has been repeated in the present study, with adjusted hazard ratios very similar to the earlier study. Also, citalopram, paroxetine, and venlafaxine were found to be significantly associated with an increased risk of epilepsy/seizures in both studies.

The significant association with sertraline in the earlier study is no longer present whereas there is now a significant association with fluoxetine. But the main difference is that in the present study we have found a significant association with TCAs when before there was none.

The previous study reported an adjusted hazard ratio of 1.02 for TCAs for older people but for the present study it was 2.32. In the previous study, none of the four TCAs were significantly associated with epilepsy/seizure risk, however in the present study they are all associated with a statistically significant increased risk with the highest hazard ratio being for trazodone.

Why TCAs should be significantly associated with an increased risk of epilepsy/seizures in the younger sample but not the older sample is unclear, particularly since the proportion of TCA prescriptions in the present study is far less than the earlier study: 31.6 % vs. 13.3 %. Although there were fewer low dose TCA prescriptions in the new study compared to the older study (60 % vs. 70 % were for ≤ 0.5 DDD), this is not enough to explain the difference between the two results.

### Strengths and limitations

We know of no other study utilising a sample as large, and followed-up for as many years as our cohort. We were able to analyse a range of antidepressant medications, including individual drugs and accounted for many confounding factors. We also calculated absolute risks that enable comparisons to be made between different drugs that can be explained to patients.

We have used a very large sample representative of primary care populations where most treatment decisions are made, so our results are likely to be generalizable to people aged 20 to 64 years with a diagnosis of depression in primary care.

Our study included all eligible patients within the study time period, and did not exclude people with any comorbid disease or taking any medications other than those explicitly stated in the exclusion criteria (patients with diagnoses of schizophrenia, bipolar disorder or other types of psychosis, and those prescribed lithium or anti-manic drugs). This limited the potential for selection bias which can affect observational studies. Recall bias was avoided as data on prescriptions and confounding factors were recorded prospectively on the computer system.

We had access to detailed information on antidepressant prescriptions, including class of antidepressant, drug name, dosage and duration, so were able to investigate associations with these factors.

To reduce the effects of bias we adjusted for many confounding factors, however we were unable to control for certain factors such as family history of seizures, seizure history without diagnosis, neurological abnormalities, or other factors known to affect seizure threshold such as antibiotic use [31–34] so there may be some residual confounding. Although we adjusted for depression severity, this was based on a logical but crude classification of diagnostic Read codes that has not been formally validated, since depression severity scores are not routinely recorded in general practice.

We excluded patients with a history of epilepsy/seizures at baseline thereby reducing the possibility of a drug being prescribed on the basis of whether it was known to affect the seizure threshold (channelling bias).

The generally similar patterns of risk for the different drug classes and individual drugs might indicate that depression itself rather than its treatment could be the cause of the increased rates of epilepsy/seizures. We restricted our cohort to patients who had a diagnosis of depression to reduce this potential indication bias however we compared rates during periods of treatment, with untreated periods when the depression may have resolved. Several studies have reported that depression is associated with increased rates of epilepsy/seizures including a bidirectional relationship [8, 16, 17], although it is difficult to fully separate the effects of depression from those of antidepressant treatment.

We do not know whether patients took their medication at the exact prescribed dose or whether they completed the course of medication. As a result, this may mean some periods of antidepressant treatment have been misclassified.

Our outcome was a diagnosis of epilepsy/seizures as recorded in the general practitioners record. The nature of the database and the study design meant that this could not be formally adjudicated as might be the case in a clinical trial. However since epilepsy is considered a major diagnosis with consequences which can affect a person’s employment or ability to drive, we think it is unlikely that it would be entered in the record without there being sufficient evidence to support the diagnosis. Also epilepsy has been a condition included in the GP Quality and Outcomes framework over recent years and this is likely to have resulted in increased diligence regarding the accuracy of the diagnosis recorded on the computer system. Another limitation of our study is that we were not able to distinguish between seizures that were epileptic and non-epileptic in nature, such as psychogenic non-epileptic seizures. There is no reason to believe however that different antidepressants would have a differential effect on inducing non-epileptic seizures.

### Implications of our findings

Whilst we acknowledge our results need further confirmation via future studies, there are possible implications of our findings for the treatment of depression.

We have found that treatment with all antidepressants is associated with an increased risk of epilepsy/seizures, with some types of antidepressant being associated with a much higher risk than others. This suggests there is a need to inform patients and their doctors about the increased risks of continued antidepressant therapy that accumulate over long periods of treatment. Consideration of each patient’s individual circumstances must be taken into account, allowing discussion between the clinician and the patient, enabling a shared decision to be reached on future treatments.

Due to the increased absolute risks of epilepsy/seizures that occur under long term use, there is a need for an individual clinical risk-benefit assessment for any patient facing antidepressant treatment over a period of five or more years, bearing in mind the absence of evidence supporting longer term treatment with antidepressants. This may be of particular importance to people with mild and infrequently recurring depression, or those with additional risk factors for seizures such as evidence of cerebrovascular disease.

In instances of mild depression in particular there may be a case for stopping treatment, or a reduced daily dosage, or switching to a different antidepressant that carries a lower risk or seeking alternative treatments, such as cognitive behavioural therapy, rather than continuing with long-term antidepressant treatment which would carry a further increased risk of epilepsy/seizures.

In instances where the choice of antidepressant and dose has already been driven by factors such as how well the patient tolerates the drug or the severity of the depression, our findings can still inform the continued treatment of the patient as the accumulating risk that occurs over time that we have demonstrated can be communicated to the patient by the clinician. An informed decision can then be reached regarding continued antidepressant treatment or alternative options from that point forwards.

## Conclusions

The increased risk to patients of epilepsy/seizures during antidepressant treatment needs to be weighed against the potential benefits of treatment, and whether the patient is on short term treatment which may not carry a clinically important increased risk or on longer term treatment where there is an accumulating risk over time. Consideration of each patient’s individual circumstances, and the choice of antidepressant, duration and dose, may play a part in deciding whether any increased risks of epilepsy/seizures are acceptable.

More research is needed to further clarify the situation with respect to the association between antidepressant use and the risk of epilepsy/seizures, taking account of the type, severity and number of seizures over time, whether patients have an existing predisposition to epilepsy/seizures, and accounting for other cofactors such as co-prescriptions, that may influence this relationship. Residual confounding and indication bias may also influence our results, so they need to be interpreted with caution.

## Additional file

Additional file 1: Table S1. List of Read codes included in the definition of the epilepsy/seizures outcome. **Table S2.** Read codes used for identification of patients with depression and their assigned severity. **Table S3.** Baseline characteristics of patients according to the class of antidepressant first prescribed. **Table S4.** 5-year hazard ratios for epilepsy/seizures by antidepressant class, dose and individual drug, with SSRIs, mid-dose SSRI and citalopram as the reference categories respectively. **Table S5.** 5-year hazard ratios for epilepsy/seizures by antidepressant class, dose and individual drug, with untreated patients excluded from the cohort. **Table S6.** 5-year hazard ratios for epilepsy/seizures by antidepressant class, dose and individual drug, with patients on anticonvulsants at baseline excluded from the cohort. **Table S7.** 1-year hazard ratios for epilepsy/seizures by antidepressant class, dose and individual drug. **Table S8.** 1-year hazard ratios for epilepsy/seizures by antidepressant class, dose and individual drug, with SSRIs, mid-dose SSRI and citalopram as the reference categories respectively. (PDF 731 kb)

## Abbreviations

BNF: British national formulary
CI: Confidence interval
DDD: Defined daily dose
EMIS: Egton medical information system
FDA: Food and drug administration
HR: Hazard ratio
MAOI: Monoamine oxidase inhibitor
NNH: Numbers needed to harm
NNT: Numbers needed to treat
SBA: Summary basis of approval
SSRI: Selective serotonin reuptake inhibitor
TCA: Tricyclic and related antidepressants
WHO: World health organisation
