# Supplementary material for: Antidepressant use and risk of epilepsy and seizures in people aged 20 to 64 years: cohort study using a primary care database
Source: BMC Psychiatry. 2015 Dec 17;15:315. doi: 10.1186/s12888-015-0701-9 (PMC4683813; doi:10.1186/s12888-015-0701-9)
Supplement: Additional file 1: Table S1. — List of Read codes included in the definition of the epilepsy/seizures outcome. Table S2. Read codes used for identification of patients with depression and their assigned severity. Table S3. Baseline characteristics of patients according to the class of antidepressant first prescribed. Table S4. 5-year hazard ratios for epilepsy/seizures by antidepressant class, dose and individual drug, with SSRIs, mid-dose SSRI and citalopram as the reference categories respectively. Table S5. 5-year hazard ratios for epilepsy/seizures by antidepressant class, dose and individual drug, with untreated patients excluded from the cohort. Table S6. 5-year hazard ratios for epilepsy/seizures by antidepressant class, dose and individual drug, with patients on anticonvulsants at baseline excluded from the cohort. Table S7. 1-year hazard ratios for epilepsy/seizures by antidepressant class, dose and individual drug. Table S8. 1-year hazard ratios for epilepsy/seizures by antidepressant class, dose and individual drug, with SSRIs, mid-dose SSRI and citalopram as the reference categories respectively. (PDF 731 kb) [file 12888_2015_701_MOESM1_ESM.pdf]

Antidepressant use and risk of epilepsy and seizures in people aged 20 to 64 years: cohort study using a primary care database

Hill T, Coupland C, Morriss R, Arthur A, Moore M, Hippisley-Cox J.

Additional file 1.pdf

Contents:

Table S1: List of Read codes included in the definition of the epilepsy/seizures outcome.

Table S2: Read codes used for identification of patients with depression and their assigned severity.

Table S3: Baseline characteristics of patients according to the class of antidepressant first prescribed.

Table S4: 5-year hazard ratios for epilepsy/seizures by antidepressant class, dose and individual drug, with SSRIs, mid-dose SSRI and citalopram as the reference categories respectively.

Table S5: 5-year hazard ratios for epilepsy/seizures by antidepressant class, dose and individual drug, with untreated patients excluded from the cohort.

Table S6: 5-year hazard ratios for epilepsy/seizures by antidepressant class, dose and individual drug, with patients on anticonvulsants at baseline excluded from the cohort.

Table S7**:** 1-year hazard ratios for epilepsy/seizures by antidepressant class, dose and individual drug.

Table S8: 1-year hazard ratios for epilepsy/seizures by antidepressant class, dose and individual drug, with SSRIs, mid-dose SSRI and citalopram as the reference categories respectively.

Table S1: List of Read codes included in the definition of the epilepsy/seizures outcome

Read term	Description		Read term	Description
F1321	Progressive myoclonic epilepsy		F2553-1	Partial epilepsy with autonomic symptoms
F25	Epilepsy		F2554	Visual reflex epilepsy
F250	Generalised non-convulsive epilepsy		F2555	Unilateral epilepsy
F2500	Petit mal (minor) epilepsy		F2556	Simple partial epileptic seizure
F2500-1	Epileptic absences		F255y	Partial epilepsy without impairment of consciousness OS
F2500-99	Petit mal epilepsy		F255z	Partial epilepsy without impairment of consciousness NOS
F2501	Pykno-epilepsy		F256	Infantile spasms
F2502	Epileptic seizures - atonic		F256-1	Lightning spasms
F2503	Epileptic seizures - akinetic		F256-2	West syndrome
F2504	Juvenile absence epilepsy		F256-99	Infantile spasms -hypsarrythmia
F2505	Lennox-Gastaut syndrome		F2560	Hypsarrhythmia
F250y	Other specified generalised non-convulsive epilepsy		F2561	Salaam attacks
F250z	Generalised non-convulsive epilepsy NOS		F256z	Infantile spasms NOS
F251	Generalised convulsive epilepsy		F257	Kojevnikov's epilepsy
F2510	Grand mal (major) epilepsy		F258	Post-ictal state
F2510-1	Tonic-clonic epilepsy		F259	Early infant epileptic encephalopathy with suppression bursts
F2510-99	Grand mal epilepsy		F259-1	Ohtahara syndrome
F2511	Neonatal myoclonic epilepsy		F25A	Juvenile myoclonic epilepsy
F2511-1	Otohara syndrome		F25B	Alcohol-induced epilepsy
F2512	Epileptic seizures - clonic		F25C	Drug-induced epilepsy
F2513	Epileptic seizures - myoclonic		F25D	Menstrual epilepsy
F2514	Epileptic seizures - tonic		F25E	Stress-induced epilepsy
F2515	Tonic-clonic epilepsy		F25F	Photosensitive epilepsy
F251y	Other specified generalised convulsive epilepsy		F25X	Status epilepticus, unspecified
F251z	Generalised convulsive epilepsy NOS		F25y	Other forms of epilepsy
F252	Petit mal status		F25y0	Cursive (running) epilepsy

F253	Grand mal status		F25y1	Gelastic epilepsy
F253-1	Status epilepticus		F25y2*	Locl-rlt(foc)(part)idiop epilep&epilptic syn seiz locl onset
F254	Partial epilepsy with impairment of consciousness		F25y3	Complex partial status epilepticus
F2540	Temporal lobe epilepsy		F25y4	Benign Rolandic epilepsy
F2541	Psychomotor epilepsy		F25yz	Other forms of epilepsy NOS
F2542	Psychosensory epilepsy		F25z	Epilepsy NOS
F2543	Limbic system epilepsy		SC200	Traumatic epilepsy
F2544	Epileptic automatism		1B27	Seizures in response to acute event
F2545	Complex partial epileptic seizure		282-3	O/E - a seizure
F2545-99	Partial complex seizure		2824-1	O/E - Jacksonian fit
F254z	Partial epilepsy with impairment of consciousness NOS		2824-2	O/E - focal fit
F255	Partial epilepsy without impairment of consciousness		282Z	O/E - fit/convulsion NOS
F2550	Jacksonian, focal or motor epilepsy		667T	Daily seizures
F2550-1	Focal epilepsy		667V	Many seizures a day
F2550-2	Motor epilepsy		EMISCTR3	Tremors/seizures
F2551	Sensory induced epilepsy		F2516	Grand mal seizure
F2552	Somatosensory epilepsy		R003z-1	[D]Seizure NOS
F2553	Visceral reflex epilepsy		F2553-1	Partial epilepsy with autonomic symptoms

* Localization-related (focal) (partial) idiopathic epilepsy and epileptic syndromes with seizures of localized onset. (ICD10 code G40.0)

Table S2: Read codes used for identification of patients with depression and their assigned severity

Read code	Read code description	Depression severity[1]
1465	H/O: depression	mild
1B17	Depressed	mild
1B17-1	C/O - feeling depressed	mild
1B1U	Symptoms of depression	mild (*)
1B1U-1	Depressive symptoms	mild (*)
1JJ	Suspected depression	mild (*)
8CAa	Patient given advice about management of depression	mild (*)
E1121	Single major depressive episode, mild	mild
E1126	Single major depressive episode, in full remission	mild
E1131	Recurrent major depressive episodes, mild	mild
E1136	Recurrent major depressive episodes, in full remission	mild
E118	Seasonal affective disorder	mild
E2003	Anxiety with depression	mild
E204	Neurotic depression reactive type	mild
E204-99	Reactive (neurotic) depression	mild (*)
E2112	Depressive personality disorder	mild
E290	Brief depressive reaction	mild
E290z	Brief depressive reaction NOS	mild (*)
E2B-98	Depression	mild (*)
E2B-99	Depression NOS	mild (*)
E2B0	Postviral depression	mild
E2B1	Chronic depression	mild
EMISCAB36	Abnormal depressed feelings	mild (*)
Eu32-1	[X]Single episode of depressive reaction	mild
Eu32-2	[X]Single episode of psychogenic depression	mild (*)

Eu32-3	[X]Single episode of reactive depression	mild (*)
Eu320	[X]Mild depressive episode	mild
Eu320-99	Mild depression	mild (*)
Eu324	[X]Mild depression	mild (*)
Eu32y	[X]Other depressive episodes	mild
Eu32y-2	[X]Single episode of masked depression NOS	mild (*)
Eu32z-1	[X]Depression NOS	mild
Eu32z-2	[X]Depressive disorder NOS	mild (*)
Eu32z-4	[X] Reactive depression NOS	mild (*)
Eu33-1	[X]Recurrent episodes of depressive reaction	mild
Eu33-2	[X]Recurrent episodes of psychogenic depression	mild
Eu33-3	[X]Recurrent episodes of reactive depression	mild (*)
Eu33-4	[X]Seasonal depressive disorder	mild (*)
Eu33-5	[X]SAD - Seasonal affective disorder	mild
Eu330	[X]Recurrent depressive disorder, current episode mild	mild
Eu341	[X]Dysthymia	mild
Eu341-1	[X]Depressive neurosis	mild
Eu341-3	[X]Neurotic depression	mild (*)
Eu3y1-1	[X]Recurrent brief depressive episodes	mild
Eu412-1	[X]Mild anxiety depression	mild
PCSDT1RE23	Reactive depression	mild (*)
R007z-3	[D]Postoperative depression	mild
2257	O/E - depressed	moderate
62T1	Puerperal depression	moderate (*)
9kQ-1	On full dose long term treatment for depression	moderate (*)
E002	Senile dementia with depressive or paranoid features	moderate
E0021	Senile dementia with depression	moderate

E002z	Senile dementia with depressive or paranoid features NOS	moderate
E112	Single major depressive episode	moderate
E112-2	Endogenous depression first episode	moderate
E112-3	Endogenous depression first episode	moderate
E1122	Single major depressive episode, moderate	moderate
E1123	Single major depressive episode, severe, without psychosis	moderate
E1125	Single major depressive episode, partial or unspec remission	moderate
E112z	Single major depressive episode NOS	moderate
E1132	Recurrent major depressive episodes, moderate	moderate
E1135	Recurrent major depressive episodes,partial/unspec remission	moderate
E1137	Recurrent depression	moderate (*)
E11y	Other and unspecified manic-depressive psychoses	moderate
E11y2	Atypical depressive disorder	moderate
E11z2	Masked depression	moderate
E204-1	Postnatal depression	moderate
E291	Prolonged depressive reaction	moderate
E2B	Depressive disorder NEC	moderate
EGTON306	Post natal depression	moderate (*)
EMISNQAN25	Antenatal depression	moderate (*)
Eu32	[X]Depressive episode	moderate (*)
Eu321	[X]Moderate depressive episode	moderate
Eu321-99	Moderate depression	moderate (*)
Eu322-3	[X]Single episode vital depression w'out psychotic symptoms	moderate (*)
Eu32B	[X]Antenatal depression	moderate (*)
Eu32y-1	[X]Atypical depression	moderate
Eu32z	[X]Depressive episode, unspecified	moderate (*)
Eu32z-3	[X]Prolonged single episode of reactive depression	moderate (*)
Eu33	[X]Recurrent depressive disorder	moderate (*)

Eu331	[X]Recurrent depressive disorder, current episode moderate	moderate (*)
Eu332-1	[X]Endogenous depression without psychotic symptoms	moderate
Eu332-2	[X]Major depression, recurrent without psychotic symptoms	moderate
Eu332-4	[X]Vital depression, recurrent without psychotic symptoms	moderate (*)
Eu334	[X]Recurrent depressive disorder, currently in remission	moderate (*)
Eu33y	[X]Other recurrent depressive disorders	moderate
Eu33z	[X]Recurrent depressive disorder, unspecified	moderate
Eu33z-1	[X]Monopolar depression NOS	moderate
Eu341-4	[X]Persistant anxiety depression	moderate
Eu3y0-1	[X]Mixed affective episode	moderate
Eu412	[X]Mixed anxiety and depressive disorder	moderate
Eu530-1	[X]Postnatal depression NOS	moderate
Eu530-2	[X]Postpartum depression NOS	moderate
HNG0531	[RFC] Postnatal depression	moderate (*)
HNGNQRF13	[RFC] Depression	moderate (*)
9kQ	On full dose long term treatment depression - enh serv admin	Severe (*)
E0013	Presenile dementia with depression	Severe
E11-2	Depressive psychoses	Severe
E112-1	Agitated depression	Severe
E112-4	Endogenous depression	Severe
E1120	Single major depressive episode, unspecified	Severe
E1124	Single major depressive episode, severe, with psychosis	Severe
E113	Recurrent major depressive episode	Severe
E113-1	Endogenous depression - recurrent	Severe
E1130	Recurrent major depressive episodes, unspecified	Severe
E1133	Recurrent major depressive episodes, severe, no psychosis	Severe
E1134	Recurrent major depressive episodes, severe, with psychosis	Severe

E113z	Recurrent major depressive episode NOS	Severe
E11y0	Unspecified manic-depressive psychoses	Severe
E130	Reactive depressive psychosis	Severe
E130-1	Psychotic reactive depression	Severe
E135	Agitated depression	Severe
Eu322	[X]Severe depressive episode without psychotic symptoms	Severe
Eu322-1	[X]Single episode agitated depressn w'out psychotic symptoms	Severe (*)
Eu322-2	[X]Single episode major depression w'out psychotic symptoms	Severe
Eu322-99	Severe depression	Severe (*)
Eu323	[X]Severe depressive episode with psychotic symptoms	Severe
Eu323-1	[X]Single episode of major depression and psychotic symptoms	Severe (*)
Eu323-2	[X]Single episode of psychogenic depressive psychosis	Severe (*)
Eu323-3	[X]Single episode of psychotic depression	Severe
Eu323-4	[X]Single episode of reactive depressive psychosis	Severe
Eu332	[X]Recurr depress disorder cur epi severe without psyc sympt	Severe (*)
Eu333	[X]Recurrent depress disorder cur epi severe with psyc symp	Severe
Eu333-1	[X]Endogenous depression with psychotic symptoms	Severe (*)
Eu333-3	[X]Recurr severe episodes/major depression+psychotic symptom	Severe
Eu333-5	[X]Recurrent severe episodes of psychotic depression	Severe
Eu333-6	[X]Recurrent severe episodes/reactive depressive psychosis	Severe

[1] Severity was assigned using codes published by Martinez and colleagues [1] plus some additional codes (*) by a member of the study team (RM).

1. Martinez C, Rietbrock S, Wise L, Ashby D, Chick J, Moseley J et al. Antidepressant treatment and the risk of fatal and non-fatal self harm in first episode depression: nested case-control study. BMJ 2005;330(7488):389.

Table S3: Baseline characteristics of patients (N=238,963) according to the class of antidepressant first prescribed

First antidepressant class prescribed[1]		No antidepressant[2]		TCA		SSRI		Other	
**Characteristic**		**n**	**%[3]**	**n**	**%[3]**	**n**	**%[3]**	**n**	**%[3]**
Gender									
	Male	11,851	40.25	12,171	38.25	64,331	38.30	4,308	47.23
	Female	17,590	59.75	19,645	61.75	103,625	61.70	4,813	52.77
Age (years)									
	20-29	7,303	24.81	5,394	16.95	36,631	21.81	1,753	19.22
	30-39	9,818	33.35	9,164	28.80	55,136	32.83	2,833	31.06
	40-49	6,328	21.49	8,286	26.04	42,105	25.07	2,370	25.98
	50-59	4,501	15.29	6,822	21.44	26,492	15.77	1,662	18.22
	60-64	1,491	5.06	2,150	6.76	7,592	4.52	503	5.51
Mean age (SD)									
	Overall	38.62 (11.35)		41.65 (11.37)		39.25 (11.00)		40.42 (11.18)	
	Male	40.18 (11.60)		42.34 (11.21)		40.89 (11.01)		40.99 (11.05)	
	Female	37.57 (11.06)		41.21 (11.45)		38.23 (10.87)		39.91 (11.28)	
Ethnicity									
	White (UK or European)	27,223	92.47	29,903	93.99	161,022	95.87	8,728	95.69
	Indian	393	1.33	282	0.89	1,189	0.71	49	0.54
	Pakistani	241	0.82	303	0.95	1,080	0.64	84	0.92
	Bangladeshi	219	0.74	230	0.72	513	0.31	36	0.39
	Other Asian	159	0.54	165	0.52	626	0.37	32	0.35
	Caribbean	330	1.12	208	0.65	927	0.55	50	0.55
	Black African	311	1.06	257	0.81	763	0.45	44	0.48
	Chinese	77	0.26	41	0.13	181	0.11	8	0.09
	Other	488	1.66	427	1.34	1,655	0.99	90	0.99
Depression severity (index diagnosis)									
	Mild	21,870	74.28	23,663	74.37	118,565	70.59	6,652	72.93
	Moderate	6,979	23.71	6,991	21.97	43,010	25.61	2,038	22.34
	Severe	592	2.01	1,162	3.65	6,39281	3.80	431	4.73

Mean BMI in kg/m^2 (SD)		26.09 (5.09)		26.90 (5.32)		26.68 (5.31)		26.52 (5.19)	
Smoking[4]									
	Recorded	28,333		31,151		164,350		8,848	
	Non smoker	14,468	51.06	14,538	46.67	77,649	47.25	3,913	44.22
	Ex smoker	4,456	15.73	4,658	14.95	24,773	15.07	1,177	13.30
	Current smoker	9,409	33.21	11,955	38.38	61,928	37.68	3,758	42.47
	Current light smoker	3,046	10.75	3,104	9.96	16,982	10.33	902	10.19
	Current moderate smoker	4,302	15.18	5,375	17.25	28,992	17.64	1,750	19.78
	Current heavy smoker	2,061	7.27	3,476	11.16	15,954	9.71	1,106	12.50
Alcohol consumption[4]									
	Recorded	24,589		27,711		142,697		7,691	
	Non drinker	6,435	26.17	8,586	30.98	37,860	26.53	2,202	28.63
	Trivial (less than 1 unit per day)	9,340	37.98	10,301	37.17	55,146	38.65	2,645	34.39
	Light (1-2 units per day)	6,607	26.87	6,404	23.11	36,215	25.38	1,957	25.45
	Moderate (3 to 6 units per day)	1,721	7.00	1,822	6.58	10,256	7.19	639	8.31
	Heavy (7 to 9 units per day)	240	0.98	251	0.91	1,559	1.09	116	1.51
	Very heavy (over 9 units per day)	246	1.00	347	1.25	1,661	1.16	132	1.72
Townsend deprivation quintile[4]									
	Recorded	28,356		30,821		162,235		8,752	
	1 (Least deprived)	5,111	18.02	5,490	17.81	32,750	20.19	1,580	18.05
	2	5,380	18.97	5,854	18.99	33,303	20.53	1,582	18.08
	3	5,589	19.71	6,170	20.02	34,703	21.39	1,725	19.71
	4	5,810	20.49	6,512	21.13	32,719	20.17	1,882	21.50
	5 (Most deprived)	6,466	22.80	6,795	22.05	28,760	17.73	1,983	22.66
Comorbidities									
	CHD	632	2.15	580	1.82	2,691	1.60	200	2.19
	Diabetes	1,195	4.06	1,145	3.60	4,761	2.83	260	2.85
	Hypertension	2,238	7.60	2,651	8.33	11,633	6.93	656	7.19
	Stroke/TIA	209	0.71	273	0.86	1,152	0.69	102	1.12

	Any cancer	403	1.37	629	1.98	2,595	1.55	167	1.83
	Epilepsy/seizures	533	1.81	394	1.24	2,125	1.27	263	2.88
	Hypothyroidism	547	1.86	786	2.47	3,728	2.22	196	2.15
	Obsessive-compulsive disorder	55	0.19	53	0.17	362	0.22	22	0.24
	Rheumatoid arthritis	118	0.40	272	0.85	846	0.50	63	0.69
	Osteoarthritis	761	2.58	1,516	4.76	4,646	2.77	284	3.11
	Osteoporosis	96	0.33	147	0.46	583	0.35	40	0.44
	Liver disease	101	0.34	119	0.37	435	0.26	40	0.44
	Renal disease	77	0.26	79	0.25	380	0.23	11	0.12
	Asthma/chronic obstructive airways disease	3,768	12.80	4,462	14.02	22,326	13.29	1,180	12.94
Medications at baseline									
	Anticonvulsants	405	1.38	453	1.42	1,524	0.91	276	3.03
	Antihypertensives	2,858	9.71	4,104	12.90	17,235	10.26	1,092	11.97
	Anti-psychotics	67	0.23	122	0.38	471	0.28	167	1.83
	Aspirin	1,005	3.41	1,147	3.61	4,664	2.78	329	3.61
	Anticoagulants	152	0.52	178	0.56	686	0.41	55	0.60
	Bisphosphonates	86	0.29	156	0.49	569	0.34	43	0.47
	Hypnotics/anxiolytics	506	1.72	1,856	5.83	8,209	4.89	735	8.06
	NSAIDs	764	2.60	3,394	10.67	7,988	4.76	542	5.94
	Statins	1,542	5.24	1,588	4.99	7,231	4.31	450	4.93
	Oral contraceptives[5]	2,975	10.10	3,043	9.56	20,577	12.25	763	8.37
	HRT[5]	590	2.00	1,450	4.56	4,886	2.91	266	2.92

[1] MAOI (n=22) and Combined group (n=607) not shown

[2] The no antidepressant column includes only patients who were never treated. For the purposes of the time-varying analyses, the no treatment exposure time included both these patients, plus periods of non-treatment for patients who did receive antidepressant treatment during follow-up.

[3] Percentages are column percentages, within each characteristic

[4] Percentages are out of the number recorded

[5] Given percentage is for female patients only

Table S4: 5-year hazard ratios for epilepsy/seizures by antidepressant class, dose and individual drug, with SSRIs, mid-dose SSRI and Citalopram as the reference categories respectively.

			Unadjusted analyses				Adjusted[3] analyses			
	No. of events[1]	Person years[2]	HR	95% CI		P	HR	95% CI		P
**Antidepressant class**										
SSRIs	309	224,600	1.00				1.00			
TCAs	82	41,130	1.44	1.12	1.83	0.004	1.21	0.94	1.56	0.147
Other antidepressants	58	27,820	1.58	1.21	2.06	0.001	1.22	0.92	1.61	0.171
Combined antidepressants	13	4,220	2.38	1.39	4.07	0.002	1.42	0.79	2.57	0.239
No current use	384	566,890	0.51	0.43	0.59	<0.001	0.52	0.44	0.61	<0.001
**Antidepressant class and dose categories[4]**										
SSRI: >0.5 DDD/≤ 1.0 DDD	213	157,490	1.00				1.00			
No current use	384	566,890	0.52	0.43	0.63	0.000	0.53	0.44	0.64	<0.001
TCA: ≤ 0.5 DDD	38	23,520	1.18	0.83	1.67	0.350	1.00	0.70	1.44	0.981
TCA: >0.5 DDD/≤ 1.0 DDD	28	8,370	2.53	1.71	3.76	0.000	1.92	1.28	2.89	0.002
TCA: > 1.0 DDD	14	5,240	1.93	1.13	3.29	0.016	1.55	0.90	2.69	0.117
SSRI: ≤ 0.5 DDD	19	15,970	0.87	0.55	1.39	0.557	0.95	0.60	1.52	0.829
SSRI: > 1.0 DDD	67	42,410	1.21	0.91	1.60	0.189	1.07	0.80	1.42	0.647
Other: ≤ 0.5 DDD	6	4,000	1.09	0.49	2.45	0.831	0.90	0.40	2.04	0.806
Other: >0.5 DDD/≤ 1.0 DDD	31	13,090	1.94	1.36	2.75	0.000	1.40	0.97	2.03	0.075
Other: > 1.0 DDD	19	8,330	1.68	1.04	2.70	0.034	1.34	0.82	2.18	0.246
Combined antidepressants	13	4,220	2.45	1.42	4.23	0.001	1.44	0.79	2.61	0.235
**Antidepressant drug**										
Citalopram (SSRI)	136	93,940	1.00				1.00			
No current use	384	566,890	0.48	0.40	0.59	<0.001	0.49	0.40	0.60	<0.001
Fluoxetine (SSRI)	111	81,780	0.93	0.72	1.22	0.615	0.94	0.72	1.23	0.666
Amitriptyline (TCA)	34	19,550	1.19	0.82	1.72	0.369	0.95	0.65	1.41	0.811

Sertraline (SSRI)	22	18,790	0.82	0.53	1.27	0.381	0.77	0.49	1.20	0.251
Venlafaxine (other)	36	15,500	1.74	1.22	2.49	0.002	1.40	0.95	2.05	0.091
Paroxetine (SSRI)	26	16,500	1.13	0.72	1.79	0.596	1.00	0.61	1.62	0.984
Mirtazapine (other)	17	10,070	1.13	0.69	1.86	0.617	0.85	0.51	1.40	0.521
Escitalopram (SSRI)	14	13,310	0.71	0.40	1.25	0.234	0.73	0.41	1.30	0.287
Dosulepin (TCA)	21	12,120	1.23	0.77	1.96	0.389	1.08	0.67	1.74	0.763
Lofepramine (TCA)	12	4,750	1.68	0.93	3.03	0.086	1.52	0.83	2.77	0.175
Trazodone (TCA)	12	2,320	3.47	1.96	6.16	<0.001	2.66	1.49	4.76	0.001
All other antidepressants	8	4,910	1.11	0.55	2.25	0.778	0.82	0.40	1.69	0.596
Combined antidepressants	13	4,220	2.27	1.32	3.88	0.003	1.33	0.73	2.41	0.345

[1] Based on numbers in adjusted analysis

[2] Person years of exposure, based on adjusted analyses

[3] Adjusted for age, sex, year of diagnosis of depression, severity of depression, deprivation, smoking status, alcohol intake, ethnic group (white/not recorded or non-white), coronary heart disease, diabetes, hypertension, cancer, hypothyroidism, osteoarthritis, asthma/chronic obstructive airways disease, stroke/TIA, rheumatoid arthritis, osteoporosis, liver disease, renal disease, obsessive-compulsive disorder, statins, NSAIDS, aspirin, antihypertensive drugs, anticonvulsants, hypnotics/anxiolytics, oral contraceptives, hormone replacement therapy, antipsychotics, bisphosphonates, anticoagulants

[4] Total numbers in the analysis of dosage are less due to missing data.

DDD = defined daily dose

Table S5: 5-year hazard ratios for epilepsy/seizures by antidepressant class, dose and individual drug, with untreated patients excluded from the cohort.

			Unadjusted analyses				Adjusted[3] analyses			
	No. of events[1]	Person years[2]	HR	95% CI		P	HR	95% CI		P
**Antidepressant class**										
No current use	310	471,190	1.00				1.00			
TCAs	82	41,130	2.92	2.28	3.74	<0.001	2.43	1.87	3.15	<0.001
SSRIs	309	224,600	2.04	1.72	2.40	<0.001	2.00	1.68	2.38	<0.001
Other antidepressants	58	27,820	3.20	2.44	4.19	<0.001	2.44	1.82	3.26	<0.001
Combined antidepressants	13	4,220	4.83	2.85	8.18	<0.001	2.86	1.59	5.14	<0.001
**Antidepressant class and dose categories[4]**										
No current use	310	471,190	1.00				1.00			
TCA: ≤ 0.5 DDD	38	23,520	2.33	1.63	3.32	<0.001	1.99	1.38	2.89	<0.001
TCA: >0.5 DDD/≤ 1.0 DDD	28	8,370	5.00	3.41	7.32	<0.001	3.82	2.58	5.65	<0.001
TCA: > 1.0 DDD	14	5,240	3.80	2.24	6.46	<0.001	3.08	1.80	5.27	<0.001
SSRI: ≤ 0.5 DDD	19	15,970	1.71	1.09	2.68	0.020	1.88	1.19	2.95	0.006
SSRI: >0.5 DDD/≤ 1.0 DDD	213	157,490	1.98	1.62	2.40	<0.001	1.98	1.62	2.42	<0.001
SSRI: > 1.0 DDD	67	42,410	2.38	1.84	3.07	<0.001	2.12	1.62	2.77	<0.001
Other: ≤ 0.5 DDD	6	4,000	2.15	0.98	4.73	0.057	1.79	0.81	3.98	0.150
Other: >0.5 DDD/≤ 1.0 DDD	31	13,090	3.81	2.70	5.39	<0.001	2.76	1.90	4.01	<0.001
Other: > 1.0 DDD	19	8,330	3.29	2.05	5.29	<0.001	2.65	1.62	4.32	<0.001
Combined antidepressants	13	4,220	4.82	2.84	8.17	<0.001	2.85	1.59	5.14	<0.001
**Antidepressant drug**										
No current use	310	471,190	1.00				1.00			
Amitriptyline (TCA)	34	19,550	2.53	1.80	3.57	<0.001	2.03	1.41	2.91	<0.001
Dosulepin (TCA)	21	12,120	2.63	1.68	4.10	<0.001	2.29	1.44	3.62	<0.001
Lofepramine (TCA)	12	4,750	3.59	2.03	6.36	<0.001	3.24	1.81	5.77	<0.001
Trazodone (TCA)	12	2,320	7.41	4.19	13.11	<0.001	5.61	3.15	9.98	<0.001

Citalopram (SSRI)	136	93,940	2.14	1.75	2.61	<0.001	2.12	1.72	2.62	<0.001
Escitalopram (SSRI)	14	13,310	1.51	0.85	2.67	0.156	1.55	0.87	2.76	0.133
Fluoxetine (SSRI)	111	81,780	2.00	1.59	2.52	<0.001	2.00	1.58	2.53	<0.001
Paroxetine (SSRI)	26	16,500	2.42	1.53	3.83	<0.001	2.12	1.31	3.42	0.002
Sertraline (SSRI)	22	18,790	1.76	1.15	2.70	0.010	1.63	1.05	2.54	0.030
Mirtazapine (other)	17	10,070	2.42	1.50	3.90	<0.001	1.78	1.09	2.91	0.021
Venlafaxine (other)	36	15,500	3.70	2.64	5.19	<0.001	2.97	2.06	4.29	<0.001
All other antidepressants	8	4,910	2.36	1.19	4.70	0.014	1.77	0.88	3.54	0.108
Combined antidepressants	13	4,220	4.83	2.85	8.18	<0.001	2.83	1.57	5.09	0.001

[1] Based on numbers in adjusted analysis

[2] Person years of exposure, based on adjusted analyses

[3] Adjusted for age, sex, year of diagnosis of depression, severity of depression, deprivation, smoking status, alcohol intake, ethnic group (white/not recorded or non-white), coronary heart disease, diabetes, hypertension, cancer, hypothyroidism, osteoarthritis, asthma/chronic obstructive airways disease, stroke/TIA, rheumatoid arthritis, osteoporosis, liver disease, renal disease, obsessive-compulsive disorder, statins, NSAIDS, aspirin, antihypertensive drugs, anticonvulsants, hypnotics/anxiolytics, oral contraceptives, hormone replacement therapy, antipsychotics, bisphosphonates, anticoagulants

[4] Total numbers in the analysis of dosage are less due to missing data on dose.

DDD = defined daily dose

Table S6: 5-year hazard ratios for epilepsy/seizures by antidepressant class, dose and individual drug, with patients on anticonvulsants at baseline excluded from the cohort.

			Unadjusted analyses				Adjusted[3] analyses			
	No. of events[1]	Person years[2]	HR	95% CI		P	HR	95% CI		P
**Antidepressant class**										
No current use	351	565,170	1.00				1.00			
TCAs	75	40,630	2.93	2.28	3.76	<0.001	2.70	2.08	3.50	<0.001
SSRIs	275	223,480	1.97	1.67	2.32	<0.001	1.96	1.65	2.32	<0.001
Other antidepressants	51	27,550	3.01	2.26	4.01	<0.001	2.52	1.86	3.41	<0.001
Combined antidepressants	12	4,120	4.88	2.82	8.46	<0.001	3.91	2.19	6.97	<0.001
**Antidepressant class and dose categories[4]**										
No current use	351	565,170	1.00				1.00			
TCA: ≤ 0.5 DDD	36	23,230	2.43	1.70	3.46	<0.001	2.35	1.63	3.38	<0.001
TCA: >0.5 DDD/≤ 1.0 DDD	23	8,270	4.52	2.94	6.95	<0.001	3.80	2.44	5.90	<0.001
TCA: > 1.0 DDD	14	5,180	4.15	2.45	7.03	<0.001	3.58	2.10	6.12	<0.001
SSRI: ≤ 0.5 DDD	16	15,900	1.57	0.96	2.58	0.074	1.74	1.06	2.87	0.029
SSRI: >0.5 DDD/≤ 1.0 DDD	192	156,780	1.94	1.61	2.35	<0.001	1.98	1.63	2.40	<0.001
SSRI: > 1.0 DDD	58	42,130	2.23	1.73	2.89	<0.001	2.02	1.54	2.63	<0.001
Other: ≤ 0.5 DDD	6	3,960	2.34	1.06	5.18	0.036	2.15	0.97	4.78	0.060
Other: >0.5 DDD/≤ 1.0 DDD	25	12,970	3.31	2.26	4.86	<0.001	2.63	1.75	3.96	<0.001
Other: > 1.0 DDD	19	8,250	3.53	2.21	5.65	<0.001	3.03	1.87	4.90	<0.001
Combined antidepressants	12	4,120	4.88	2.82	8.46	<0.001	3.92	2.20	6.99	<0.001
**Antidepressant drug**										
No current use	351	565,170	1.00				1.00			
Amitriptyline (TCA)	30	19,220	2.45	1.70	3.52	<0.001	2.31	1.59	3.34	<0.001
Dosulepin (TCA)	20	12,050	2.74	1.76	4.26	<0.001	2.48	1.57	3.90	<0.001
Lofepramine (TCA)	11	4,720	3.61	1.99	6.56	<0.001	3.26	1.78	5.98	<0.001

Trazodone (TCA)	11	2,300	7.40	4.05	13.51	<0.001	5.95	3.26	10.85	<0.001
Citalopram (SSRI)	120	93,440	2.04	1.66	2.51	<0.001	2.05	1.65	2.54	<0.001
Escitalopram (SSRI)	11	13,240	1.29	0.68	2.46	0.439	1.33	0.69	2.54	0.391
Fluoxetine (SSRI)	100	81,410	1.96	1.55	2.46	<0.001	1.99	1.57	2.52	<0.001
Paroxetine (SSRI)	23	16,450	2.36	1.52	3.66	<0.001	2.08	1.31	3.31	0.002
Sertraline (SSRI)	21	18,670	1.83	1.20	2.79	0.005	1.74	1.13	2.69	0.012
Mirtazapine (other)	16	9,970	2.47	1.51	4.03	<0.001	1.95	1.18	3.21	0.009
Venlafaxine (other)	31	15,370	3.40	2.37	4.87	<0.001	2.96	2.02	4.35	<0.001
All other antidepressants	7	4,820	2.26	1.09	4.69	0.028	2.17	1.05	4.47	0.036
Combined antidepressants	12	4,120	4.88	2.82	8.46	<0.001	3.90	2.18	6.95	<0.001

[1] Based on numbers in adjusted analysis

[2] Person years of exposure, based on adjusted analyses

[3] Adjusted for age, sex, year of diagnosis of depression, severity of depression, deprivation, smoking status, alcohol intake, ethnic group (white/not recorded or non-white), coronary heart disease, diabetes, hypertension, cancer, hypothyroidism, osteoarthritis, asthma/chronic obstructive airways disease, stroke/TIA, rheumatoid arthritis, osteoporosis, liver disease, renal disease, obsessive-compulsive disorder, statins, NSAIDS, aspirin, antihypertensive drugs, anticonvulsants, hypnotics/anxiolytics, oral contraceptives, hormone replacement therapy, antipsychotics, bisphosphonates, anticoagulants

[4] Total numbers in the analysis of dosage are less due to missing data on dose.

DDD = defined daily dose

Table S7: 1-year hazard ratios for epilepsy/seizures by antidepressant class, dose and individual drug.

			Unadjusted analyses				Adjusted[3] analyses			
	No. of events[1]	Person years[2]	HR	95% CI		P	HR	95% CI		P
**Antidepressant class**										
No current use	80	94,330	1.00				1.00			
TCAs	30	16,340	2.11	1.35	3.30	0.001	1.77	1.11	2.83	0.016
SSRIs	129	99,590	1.54	1.14	2.06	0.004	1.55	1.15	2.10	0.004
Other antidepressants	18	8,190	2.79	1.72	4.52	<0.001	1.99	1.18	3.36	0.010
Combined antidepressants	7	860	10.73	5.09	22.62	<0.001	6.13	2.58	14.58	<0.001
**Antidepressant class and dose categories[4]**										
No current use	80	94,330	1.00				1.00			
TCA: ≤ 0.5 DDD	12	9,520	1.43	0.75	2.70	0.277	1.21	0.62	2.33	0.580
TCA: >0.5 DDD/≤ 1.0 DDD	14	3,030	5.22	2.91	9.37	<0.001	4.19	2.31	7.61	<0.001
TCA: > 1.0 DDD	3	1,990	1.68	0.54	5.24	0.368	1.47	0.47	4.58	0.511
SSRI: ≤ 0.5 DDD	9	6,090	1.68	0.84	3.36	0.143	1.76	0.87	3.56	0.117
SSRI: >0.5 DDD/≤ 1.0 DDD	94	77,030	1.42	1.03	1.97	0.032	1.46	1.05	2.02	0.023
SSRI: > 1.0 DDD	21	12,950	1.86	1.14	3.02	0.013	1.81	1.10	2.97	0.020
Other: ≤ 0.5 DDD	2	1,260	1.79	0.45	7.20	0.411	1.59	0.39	6.42	0.518
Other: >0.5 DDD/≤ 1.0 DDD	12	4,350	3.63	2.07	6.36	<0.001	2.57	1.40	4.74	0.002
Other: > 1.0 DDD	3	1,640	2.10	0.67	6.65	0.205	1.53	0.46	5.09	0.486
Combined antidepressants	7	860	10.68	5.06	22.54	<0.001	6.05	2.53	14.46	<0.001
**Antidepressant drug**										
No current use	80	94,330	1.00				1.00			
Citalopram (SSRI)	66	40,630	1.90	1.35	2.66	<0.001	1.99	1.40	2.81	<0.001
Fluoxetine (SSRI)	44	38,180	1.36	0.94	1.98	0.106	1.41	0.96	2.07	0.078
Amitriptyline (TCA)	14	6,920	2.33	1.30	4.18	0.005	1.84	1.00	3.36	0.049
Sertraline (SSRI)	5	7,550	0.91	0.39	2.09	0.820	0.76	0.31	1.88	0.557

Venlafaxine (other)	11	4,260	3.49	1.96	6.21	<0.001	2.49	1.30	4.76	0.006
Paroxetine (SSRI)	8	7,850	1.31	0.65	2.63	0.446	1.06	0.50	2.24	0.877
Mirtazapine (other)	5	3,210	1.77	0.73	4.30	0.209	1.38	0.55	3.42	0.493
Escitalopram (SSRI)	6	5,300	1.29	0.56	3.01	0.549	1.37	0.59	3.21	0.468
Dosulepin (TCA)	9	5,510	1.88	0.93	3.81	0.080	1.71	0.83	3.50	0.144
Lofepramine (TCA)	3	2,280	1.48	0.47	4.63	0.498	1.43	0.46	4.51	0.538
Trazodone (TCA)	3	820	4.16	1.30	13.34	0.016	2.85	0.79	10.21	0.108
All other antidepressants	3	1,620	2.12	0.65	6.99	0.215	1.47	0.44	4.85	0.530
Combined antidepressants	7	860	10.73	5.09	22.61	<0.001	6.16	2.59	14.64	<0.001

[1] Based on numbers in adjusted analysis

[2] Person years of exposure, based on adjusted analyses

[3] Adjusted for age, sex, year of diagnosis of depression, severity of depression, deprivation, smoking status, alcohol intake, ethnic group (white/not recorded or non-white), coronary heart disease, diabetes, hypertension, cancer, hypothyroidism, osteoarthritis, asthma/chronic obstructive airways disease, stroke/TIA, rheumatoid arthritis, osteoporosis, liver disease, renal disease, obsessive-compulsive disorder, statins, NSAIDS, aspirin, antihypertensive drugs, anticonvulsants, hypnotics/anxiolytics, oral contraceptives, hormone replacement therapy, antipsychotics, bisphosphonates, anticoagulants

[4] Total numbers in the analysis of dosage are less due to missing data.

DDD = defined daily dose

Table S8: 1-year hazard ratios for epilepsy/seizures by antidepressant class, dose and individual drug, with SSRIs, mid-dose SSRI and Citalopram as the reference categories respectively.

			Unadjusted analyses				Adjusted[3] analyses			
	No. of events[1]	Person years[2]	HR	95% CI		P	HR	95% CI		P
**Antidepressant class**										
SSRIs	129	99,950	1.00				1.00			
TCAs	30	16,340	1.37	0.93	2.03	0.114	1.14	0.76	1.72	0.523
Other antidepressants	18	8,190	1.81	1.14	2.90	0.013	1.28	0.78	2.12	0.333
Combined antidepressants	7	860	6.99	3.37	14.48	<0.001	3.95	1.70	9.17	0.001
No current use	80	94,330	0.65	0.48	0.87	0.004	0.64	0.48	0.87	0.004
**Antidepressant class and dose categories[4]**										
SSRI: >0.5 DDD/≤ 1.0 DDD	94	77,030	1.00				1.00			
No current use	80	94,330	0.70	0.51	0.97	0.032	0.69	0.49	0.95	0.023
TCA: ≤ 0.5 DDD	12	9,520	1.00	0.56	1.78	0.999	0.83	0.45	1.51	0.533
TCA: >0.5 DDD/≤ 1.0 DDD	14	3,030	3.67	2.07	6.50	<0.001	2.87	1.60	5.16	<0.001
TCA: > 1.0 DDD	3	1,990	1.18	0.38	3.71	0.775	1.00	0.32	3.17	0.994
SSRI: ≤ 0.5 DDD	9	6,090	1.18	0.59	2.36	0.641	1.21	0.60	2.43	0.601
SSRI: > 1.0 DDD	21	12,950	1.30	0.80	2.13	0.289	1.24	0.75	2.03	0.401
Other: ≤ 0.5 DDD	2	1,260	1.26	0.31	5.09	0.748	1.09	0.27	4.41	0.907
Other: >0.5 DDD/≤ 1.0 DDD	12	4,350	2.55	1.45	4.48	0.001	1.76	0.95	3.26	0.070
Other: > 1.0 DDD	3	1,640	1.48	0.47	4.69	0.508	1.05	0.32	3.48	0.937
Combined antidepressants	7	860	7.50	3.59	15.64	<0.001	4.14	1.76	9.76	0.001
**Antidepressant drug**										
Citalopram (SSRI)	66	40,630	1.00				1.00			
No current use	80	94,330	0.53	0.38	0.74	<0.001	0.50	0.36	0.71	<0.001
Fluoxetine (SSRI)	44	38,180	0.72	0.49	1.05	0.084	0.71	0.49	1.04	0.079
Amitriptyline (TCA)	14	6,920	1.23	0.69	2.19	0.482	0.92	0.51	1.68	0.795

Sertraline (SSRI)	5	7,550	0.48	0.21	1.10	0.084	0.38	0.15	0.95	0.039
Venlafaxine (other)	11	4,260	1.84	1.02	3.34	0.044	1.25	0.64	2.43	0.509
Paroxetine (SSRI)	8	7,850	0.69	0.34	1.39	0.299	0.53	0.25	1.14	0.105
Mirtazapine (other)	5	3,210	0.93	0.38	2.31	0.881	0.69	0.27	1.75	0.435
Escitalopram (SSRI)	6	5,300	0.68	0.30	1.58	0.373	0.69	0.30	1.59	0.385
Dosulepin (TCA)	9	5,510	0.99	0.50	1.95	0.982	0.86	0.43	1.71	0.664
Lofepramine (TCA)	3	2,280	0.78	0.24	2.51	0.680	0.72	0.22	2.33	0.585
Trazodone (TCA)	3	820	2.20	0.70	6.91	0.178	1.43	0.40	5.13	0.580
All other antidepressants	3	1,620	1.12	0.35	3.63	0.849	0.74	0.22	2.42	0.616
Combined antidepressants	7	860	5.66	2.69	11.91	<0.001	3.10	1.31	7.32	0.010

[1] Based on numbers in adjusted analysis

[2] Person years of exposure, based on adjusted analyses

[3] Adjusted for age, sex, year of diagnosis of depression, severity of depression, deprivation, smoking status, alcohol intake, ethnic group (white/not recorded or non-white), coronary heart disease, diabetes, hypertension, cancer, hypothyroidism, osteoarthritis, asthma/chronic obstructive airways disease, stroke/TIA, rheumatoid arthritis, osteoporosis, liver disease, renal disease, obsessive-compulsive disorder, statins, NSAIDS, aspirin, antihypertensive drugs, anticonvulsants, hypnotics/anxiolytics, oral contraceptives, hormone replacement therapy, antipsychotics, bisphosphonates, anticoagulants

[4] Total numbers in the analysis of dosage are less due to missing data.

DDD = defined daily dose
